# Scalable Fabrication of Stainless Steel–CuO Hybrid Nanostructured Electrodes for Nonenzymatic Glucose Sensing

**DOI:** 10.1021/acsomega.6c08394

**Published:** 2026-10-02

**Authors:** Kelly T. C. Thomaz, Jordelson A. A. Junior, Raphael M. V. Rocha, Fernando I. L. Leite, William Reis de Araujo, Andrei Alaferdov

**Affiliations:** 1 Laboratório de Sensores Químicos Portáteis, Instituto de Química, 28132 Universidade Estadual de Campinas, Campinas, SP 13083-862, Brazil; 2 Department of Hardware and Microelectronics, Instituto de Pesquisas Eldorado, Campinas, SP 13083-898, Brazil; 3 Faculty of Electrical and Computer Engineering, 28132 Universidade Estadual de Campinas, Campinas, SP 13083-852, Brazil; 4 Center for Semiconductor Components and Nanotechnologies, Universidade Estadual de Campinas, Campinas 13083-870, Brazil

## Abstract

A hybrid nanostructure electrode consisting of stainless-steel films decorated with copper oxide nanoparticles (CuONPs) was fabricated via direct current (DC) sputtering followed by thermal oxidation, and its structural, chemical, and electrochemical properties were systematically investigated. The nanoscale morphology and structure were examined by scanning electron microscopy (SEM) and transmission electron microscopy (TEM), including selected area electron diffraction (SAED), revealing well-defined copper nanostructures anchored to the stainless-steel surface. X-ray diffraction (XRD) and X-ray photoelectron spectroscopy (XPS) confirmed the high crystallinity of the CuONPs and the coexistence of Cu^+^ and Cu^2+^ species (Cu_2_O, CuO, Cu­(OH)_2_). Electrochemical studies in an alkaline medium demonstrated that the Cu^+^/Cu^2+^ active layer exhibits significant electrocatalytic activity toward glucose oxidation, producing a pronounced anodic response around 0.6 V vs carbon pseudoreference. The combination of crystalline CuONPs and a robust stainless-steel support, fabricated via a scalable process, makes this hybrid structure a promising candidate for nonenzymatic glucose detection.

## Graphical abstract

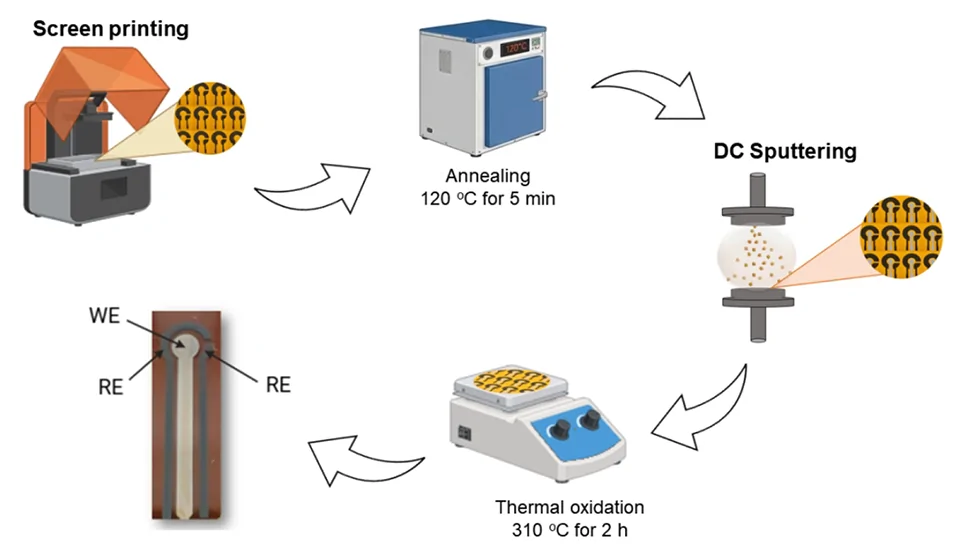

## 1 Introduction

The continuous monitoring of glucose levels plays a pivotal role in the precise diagnosis and long-term management of diabetes mellitus, one of the most prevalent metabolic disorders worldwide that remains a critical public health concern.^1,2^ In healthy individuals, fasting blood glucose levels typically range between 70 and 99 mg/dL. Values between 100 and 125 mg/dL are classified as impaired fasting glucose or prediabetes, a condition associated with an increased risk of progression to diabetes mellitus.^3^ In patients with diabetes, particularly those with type 1 and type 2 diabetes mellitus, continuous glucose monitoring is essential to maintain glycemic levels within clinically recommended target ranges, thereby minimizing the risk of acute and long-term complications.

Traditionally, glucose detection relies on enzymatic sensors employing glucose oxidase or glucose dehydrogenase due to their high specificity and sensitivity.^4,5^ Despite their outstanding analytical performance, enzymatic sensors suffer from drawbacks, such as enzyme denaturation and complex immobilization procedures. In addition, enzyme-based systems are susceptible to environmental factors such as temperature, pH, and humidity, which can significantly affect sensor lifespan.^6,7^ These challenges, coupled with the relatively high fabrication cost of such devices, have driven the development of nonenzymatic electrochemical glucose sensors as a more economically viable alternative. Such systems rely on the direct electrocatalytic oxidation of glucose on the surface of a catalytic electrode, typically composed of transition metals, metal oxides, or hybrid materials.^8,9^

Nonenzymatic electrochemical sensors are particularly attractive owing to their high sensitivity, good selectivity, long-term stability, and relatively low fabrication cost.^6,10^ In recent years, a wide range of electrocatalytic materials have been investigated for this purpose, including metals, metal oxides, polymers and carbon nanomaterials.^11–16^ Despite significant progress in this field, the practical and large-scale implementation of nonenzymatic glucose sensors still depends on several critical factors, such as a low price, physicochemical stability to enable adequate shelf life, robustness under real operating conditions, and the standardization of fabrication processes.^5,8,17^ Furthermore, according to the ASSURED criteria proposed by the World Health Organization, ideal diagnostic tools should be Affordable, Sensitive, Specific, User-friendly, Rapid and robust, Equipment-free, and Deliverable to end users.^18^ Therefore, the development of new glucose detection systems that meet these requirements represents an important step toward the implementation of point-of-care diagnostics.

Among the wide variety of materials investigated for glucose-sensitive electrochemical platforms, noble metals such as Au, Pd and Pt,^19–21^ as well as transition metals including Ni, Zn, Cu and Co, and their corresponding oxides,^22–24^ have been extensively explored. Electrodes based on noble metals exhibit high catalytic activity and good stability; however, their high production cost and susceptibility to surface poisoning significantly limit their practical applications. Consequently, systems employing transition metals emerge as excellent candidates for this purpose, as they combine effective electrocatalytic activity with improved cost-effectiveness. Copper is a well-known metal for its high electrocatalytic efficiency toward glucose oxidation.^25,26^

The electrocatalytic activity of Cu-based materials toward glucose oxidation is strongly influenced by their surface redox chemistry, particularly the reversible transformation between Cu^2+^ and Cu^3+^ species, which are widely recognized as the primary active centers for glucose electrooxidation.^27^ Under alkaline conditions, Cu surfaces undergo electrochemical oxidation, forming higher-valence species such as CuOOH or Cu^3+^-based oxyhydroxides, which mediate glucose oxidation. However, the generation of these catalysts requires specific operational conditions, including strongly alkaline electrolytes or electrochemical surface activation steps, such as potential cycling or anodic polarization.^27,28^ Various copper-based nanostructures and metal-oxide composites have demonstrated outstanding performance, particularly when exhibiting nanostructured configuration that increase the electroactive surface area and enhance electron transfer during the glucose oxidation process.^29–31^

Regarding the fabrication of electrochemical sensors, significant progress has been achieved in recent years owing to advances in deposition technologies, which have enabled the development of increasingly miniaturized, stable and highly reproducible platforms. Among the most relevant fabrication approaches are 3D printing, laser printing, screen printing and sputtering.^32,33^ 3D printing allows for rapid production of customized sensors and direct integration into wearable or microfluidic devices. However, this technique still faces certain challenges, such as nonscalability, the relatively high electrical resistance of conductive filaments, the need for postprocessing steps to partially remove nonconductive materials, and limitations in printing resolution, which directly affect the sensitivity and reproducibility of the fabricated devices.^34^ Laser printing has emerged as a particularly attractive method for wearable sensors and integrated analytical platforms; nonetheless, morphological control strongly depends on modified polymer properties and laser parameters, such as power, and uniformity remains a challenge, in addition to being a costly technique.^34^ On the other hand, screen printing is one of the most established techniques for the fabrication of portable and disposable sensors, owing to its scalability, simplicity, low cost, and moderate to high reproducibility. Sputtering enables the deposition of thin films as well as nanostructures of metal, oxides, and semiconductors with high purity, adhesion, and nanometric thickness control, promoting the formation of active surfaces with enhanced electrocatalytic properties.^35^ Despite a relatively high equipment cost, this technique offers superior precision and reproducibility, making it highly suitable for the fabrication of high-performance electrochemical sensors. Along with this, the relatively high costs associated with the sputtering technique can be mitigated by employing industrial-scale machinery, thereby enabling a more scalable and cost-effective fabrication process.

Thus, the combination of sputtering and screen-printing techniques has emerged as a promising approach for the fabrication of high-performance electrochemical sensors, owing to their ability to produce low-cost electrodes with excellent reproducibility, adhesion and tuned morphology. The integration of these techniques results in hybrid devices that combine the robustness and stability of sputtered films with the versatility and simplicity of the screen-printing process.^36,37^ In this study, we developed a hybrid electrochemical sensor composed of a stainless steel (SS) film decorated with copper oxide nanoparticles (CuONPs), assembled on a polymeric polyimide (PI) substrate using magnetron DC sputtering and low-temperature thermal treatment techniques. The sputter deposition provided a scalable approach with precise control over film thickness and enabled the formation of structures with homogeneous surface morphology, whereas low-temperature thermal treatment allowed the fabrication process to remain cost-effective. The implementation of a hybrid structure of SS/CuONPs offered a trade-off between durability and catalytic efficiency, while maintaining economic viability. The development of such a hybrid device, fabricated through reproducible and scalable techniques, represents a strategic route toward the production of sensitive and selective nonenzymatic glucose sensors with strong potential for integration into portable clinical diabetes monitoring.

## 2 Experimental Section

### 2.1 Materials and Reagents

The standards of hexaammine­ruthenium­(III) chloride [Ru­(NH_3_)_6_]­Cl_3_, NaOH, d-glucose (≥99.5% purity), d-fructose (≥99% purity), uric acid (≥99% purity), ascorbic acid (99% purity) and dopamine hydrochloride (≥98% purity) were purchased from Sigma-Aldrich, Na_2_SO_4_ (≥99% purity) was purchased from Merck. All reagents were of analytical grade, and all solutions were prepared in ultrapure water (ρ ≥ 18.2 MΩ cm) obtained using a Milli-Q system. A glucose stock solution at 0.02 mol L^–1^ was prepared daily in a 0.1 mol L^–1^ NaOH aqueous solution, which was also used as the supporting electrolyte.

### 2.2 Electrode Fabrication

A 3-electrode setup was fabricated on a flexible polyimide substrate, combining the screen-printing technique for the deposition of carbon ink on the pseudoreference and counter electrodes, and the sputtering technique for the deposition of the electroactive material on the working electrode. The electroactive material consists of a hybrid structure composed of a stainless-steel film decorated with copper oxide nanoparticles, deposited by sputtering and subsequently subjected to thermal treatment.

The manufacturing process of the sensor prototype was divided into two main steps: (i) fabrication of the reference and counter electrodes using the screen-printing method (US-2000X, ESE), and (ii) deposition of the working electrode employing a DC sputtering technique (21-LAT-001, LAT), followed by thermal oxidation (Figure 1). The printed electrodes were fabricated on a PI substrate (HSF095G, Eleven Electron) with a thickness of 95 μm and a diameter of 19 cm, using a stencil containing 44 sensor prototype structures. Printing of each substrate was performed in a single squeegee pass at a velocity of 30 mm s^–1^ under a pressure of 4 kgf. For the fabrication of 15 substrates, approximately 50 mL of carbon ink (EDAG 423SS, Loctite) was consumed. The printed structures were subsequently annealed at 120 °C for 5 min.

**Figure 1:**
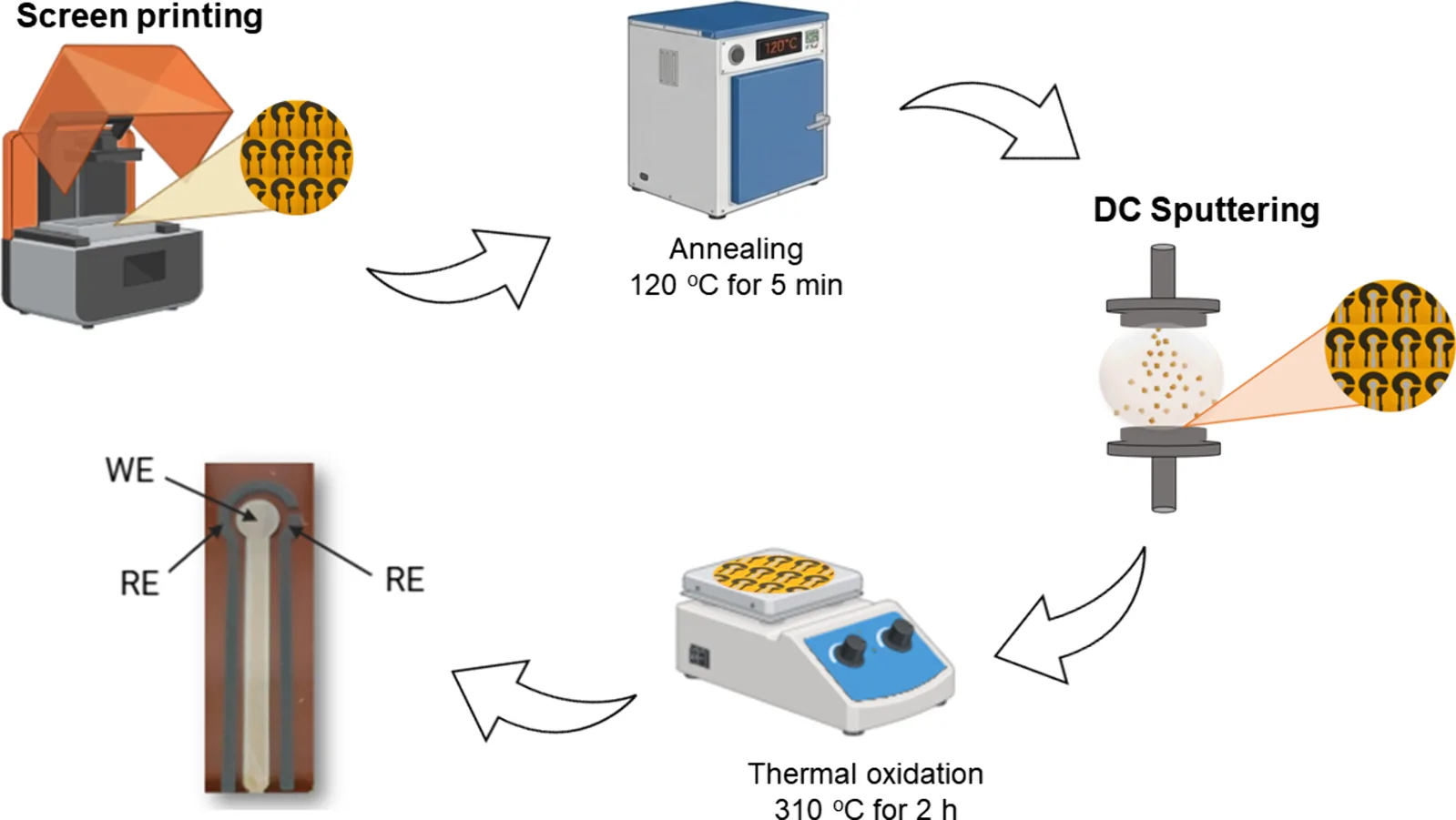
Schematic representation of the main steps involved in the device fabrication by screen printing and sputtering. This image created with BioRender.

The DC sputtering was carried out using a dual-chamber magnetron system equipped with a 254 mm diameter copper target (99.997% purity) and a stainless steel 316 target employing a schedule mask (details of the sputtering process can also be found in ref 38). The sputtering process was conducted at room temperature and under a pressure of (4–6) × 10^–4^ mbar, using argon (99.999% purity) as the working gas. The fabrication of the thin film constituting the working electrode was performed in two stages. In the first chamber, a stainless-steel film was sputtered under an argon flow rate of 15 sccm for 25 s, with a power of 2 kW for 700 s and a target–substrate distance of 81 mm. In the second chamber, Cu nanoparticles were deposited under an argon flow rate of 20 sccm for 100 s, with a power of 3 kW for 6 s and a target–substrate distance of 90 mm. The thermal oxidation of the resulting hybrid structure was performed at 310 °C for 2 h on a hot plate.

### 2.3 Characterizations of Fabricated Samples

The investigation of the fabricated samples’ morphology was carried out using scanning electron microscopy—SEM (JSM-IT200, JEOL) equipped with a silicon-drift energy dispersive spectroscopy (EDS) detector, as well as high-resolution SEM – HR SEM mode in dual beam system (Nova 200 Nanolab, FEI). Elemental mapping and chemical analyses were conducted via EDS by scanning areas of approximately 100 μm^2^ at a minimum of three distinct regions of each sample to ensure representative results. The surface atomic composition of the samples was determined using an X-ray photoelectron spectroscopy—XPS (K-Alpha model, Thermo Fisher) equipped with an ultrahigh vacuum chamber at 10^–9^ Pa and a monochromated, microfocused, low-power Al Kα X-ray source (1486.6 eV) radiation as the excitation source. The peak-fitting analysis of all photoemission spectra was accomplished using CasaXPS software (2.3.14). The X-ray diffraction—XRD (X ′Pert-MPD, Philips Analytical) was employed to identify the crystalline phases of the samples. The data were obtained using Cu Kα (1.54 Å) radiation source and a standard configuration in Bragg–Brentano geometry, within the 2θ range of 30° - 80° with a scanning rate of 0.02° s^–1^.

Detailed study of the structure of CuONPs deposited onto holey carbon film supported gold TEM grids (HC400-Au-100, EMS), as well as analysis of their crystalline phases, was performed using high-resolution transmission electron microscopy—HR TEM (JEM-2100F, JEOL) operated at 200 kV, in combination with select area electron diffraction technique—SAED. Crystalline phase analysis was carried out both for nanoparticle arrays, using SAED (analyzed area varied from 0.01 to 0.1 mm^2^), and for individual nanoparticles, by applying Fast Fourier transform (FFT) to HR TEM images for each particle. A custom Python pipeline was used to analyze HR TEM, FFT and SAED images (see details in Section S1 of the Supporting Information).

### 2.4 Electrochemical Measurements

The electrochemical measurements were performed using an AUTOLAB PGSTAT-30 potentiostat/galvanostat (Metrohm) interfaced with a computer and managed by NOVA 2.1 software. The measurements were performed using different disposable electrodes by dropping 50 μL of the sample on the sensor testing area. The electrochemical behavior of the sensor and its sensitivity toward glucose detection were investigated by cyclic voltammetry (CV) using a 3 mmol L^–1^ glucose in 0.1 mol L^–1^ NaOH solution as the supporting electrolyte, within a potential window from −0.6 to 0.7 V at a scan rate of 20 mV s^–1^. Glucose determination was performed using amperometric measurements, applying a potential of 0.6 V for 60 s. All measurements were performed in triplicate (n = 3) using three different electrodes, and five glucose concentrations ranging from 3 to 11 mmol L^–1^ were tested.

Selectivity tests were performed by cyclic voltammetry at a scan rate of 20 mV s^–1^, considering the current response at 0.6 V, at the same experimental conditions reported above, evaluating the individual concentrations of 5 mmol L^–1^ glucose, 0.5 mmol L^–1^ of each potential interfering species (uric acid, ascorbic acid, dopamine and fructose), as well as a mixture of 5 mmol L^–1^ glucose and 0.5 mmol L^–1^ of each potential interfering species.

## 3 Results and Descussion

The fabricated printed carbon electrodes with thickness of 30 ± 2 μm show excellent electrical conductivity of about 0.1–0.3 S cm^–1^, which is required for efficient electron transfer and enhanced electrochemical performance during glucose oxidation.

Investigation of the SS/CuONPs composites by SEM and TEM techniques (Figure 2) reveals a dense granular structure composed of faceted surface SS grains with a lateral size of 364 ± 19 nm forming a crack-free, continuous, and uniform layer with good adhesion to substrate. It should be noted that this granular nonflat morphology provides an enlarged effective surface area capable of accommodating a higher density of CuONPs. Detailed study of the hybrid structure morphology shows that the CuONPs with a lateral size of 11 ± 1 nm uniformly cover the facets of the SS grains (Figure 2a). The EDS mapping analysis distinctly reveals the spatial distribution of the constituent elements throughout the hybrid system, confirming their successful incorporation and homogeneous dispersion within the electrode. As illustrated in the inset images of Figure 2a and Figure 2b, the results confirm the uniform distribution of copper oxide nanoparticles and the consistent presence of SS elements across the fabricated hybrid structure. An almost perfect atomic arrangement is observed in the HR TEM images, together with the reflex structure of the SAED patterns, indicating the high crystallinity of the fabricated CuONPs (Figures 2c and 2d). Crystalline structures typically provide higher electrical conductivity and structural stability, which are beneficial for efficient electron transfer during electrochemical reactions.^39^ Nonflat morphology of the SS film, combined with relatively dense and uniform coverage of CuONPs on the SS grain facets, yielded an enlarged effective surface area of the working electrode, which increases the number of accessible catalytic sites and, consequently, enhances the electrocatalytic performance of the hybrid material.

**Figure 2:**
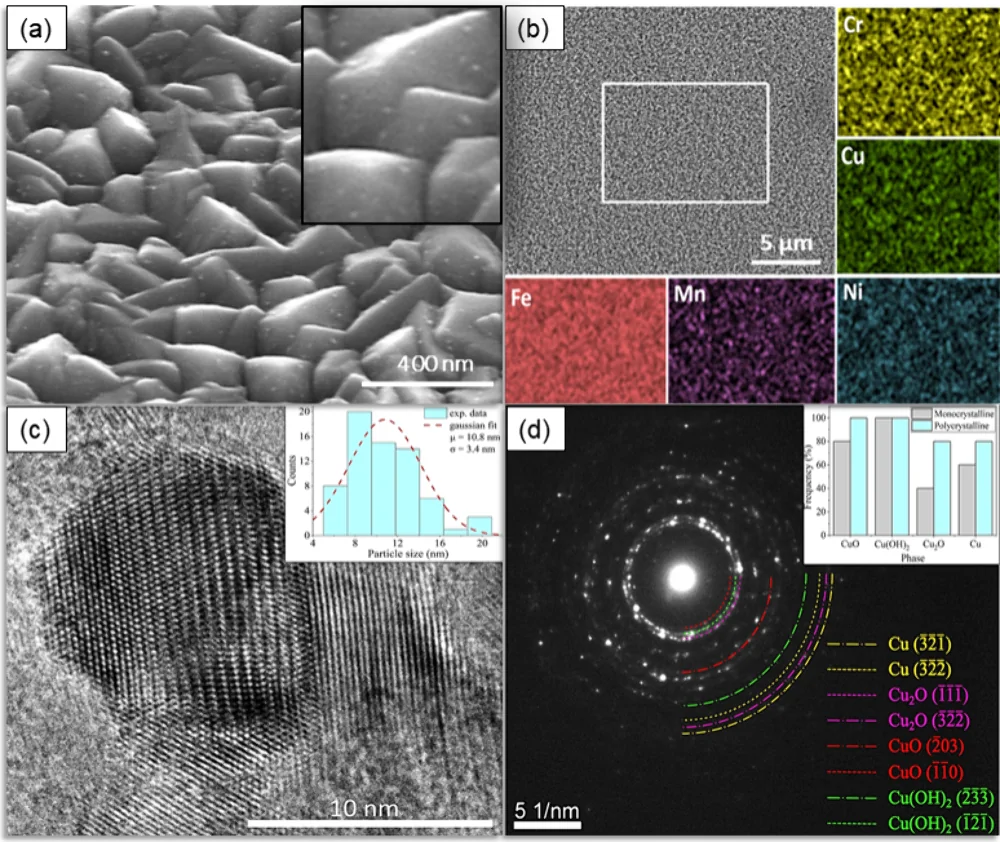
Structural and microstructural characterization of the sample: (a) HRSEM image of stainless steel grain decorated by CuONPs; the inset image highlights the uniform coverage of SS grains by CuONPs; (b) EDS elemental mapping of the region highlighted by the white rectangle in the SEM image, showing the spatial distribution of Cr, Cu, Fe, Mn, and Ni; (c) HR TEM image revealing well-defined lattice fringes, indicative of crystalline domains; the inset shows particle size distribution histogram fitted with a normal distribution, together with the experimental size profile. Statistical metrics, including median, standard deviation, and the 95% confidence interval of the mean, are summarized in the inset. (d) The typical SAED pattern obtained from CuONPs array; the inset shows the frequency of occurrence of each copper phase across five analyzed SAED patterns.

Analysis of FFT patterns obtained from 59 individual copper nanoparticles shows that approximately four-fifths of the particles contain Cu^2+^ species in either CuO and Cu­(OH)_2_ configurations. Within this fraction, nearly half exhibit exclusively CuO (∼12%), Cu­(OH)_2_ (∼12%), or mixed CuO + Cu­(OH)_2_ (∼24%) phases. The remaining half of the Cu^2+^ sites are distributed across multiple configurations, including Cu^2+^ + Cu^0^, Cu^2+^ + Cu^1+^, and Cu^2+^ + Cu^1+^ + Cu^0^ (Figure 3). The presence of the metallic phase in more than one-quarter of the particles and of the Cu^1+^ phase in two-fifths of the particles, combined with the coexistence of Cu^0^, Cu^1+^, and Cu^2+^ phases, suggests an oxidation mechanism driven by local oxygen chemical potential gradients. This process favors the formation of core–shell-like nanostructures with partially oxidized/no oxidized internal domains.^40–42^ Results of SAED studying performed on five different regions of sample (see an example in the inset of Figure 2c) corroborate first conclusion obtained from FFT analysis of individual NPs: (i) Cu^2+^, represented by both monocrystalline and polycrystalline CuO and Cu­(OH)_2_ phases, constitutes the most common sites among all nanoparticles; (ii) Cu^0^ and Cu^1+^ states, predominantly represented by polycrystalline ordering, are also widely observed, in contrast to the monocrystalline configuration of this phase, which is rarely find in nanoparticles. This experimental observation further suggests the formation of core–shell-like structures with partially oxidized internal regions. Thus, despite the part of the nanoparticles composed solely of the CuO phase—which is particularly relevant for glucose electrooxidation, acting as the primary electrochemically active sites for the reaction^43^—being relatively low among the analyzed particles, the frequent occurrence of CuO in combination with other phases ensures that the nanoparticle array remains effective for glucose electrooxidation.

**Figure 3:**
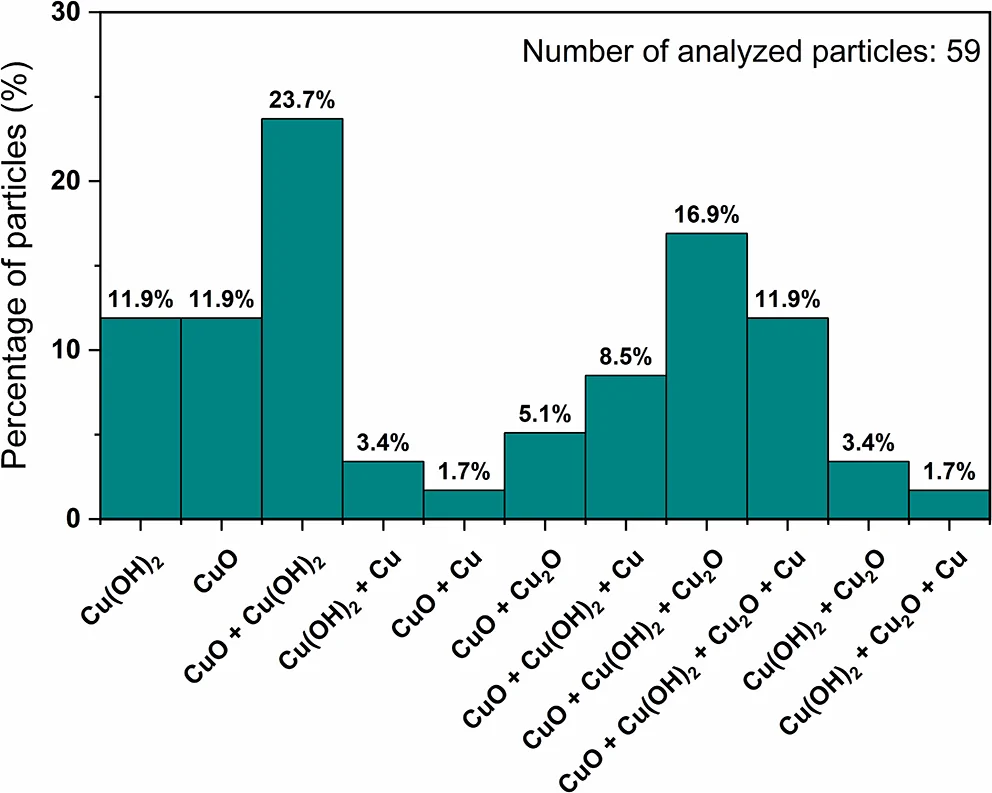
Quantitative FFT analysis results of HR TEM images of individual NPs: identified phases and phase combinations observed within the CuONPs.

Figure 4a also shows the diffraction patterns of three samples after the thermal treatment procedure: the SS thin film, the bulk copper film, and the hybrid SS/CuONPs structure. As can be seen, the conducted layer of hybrid structure exhibits the same crystalline arrangement as pure SS films, which is evidenced by the pronounced peak localized at 44.74° (2θ) and a low-intensity peak near 65.07° (2θ), usually associated with the martensite ά-phase of SS.^44^ The narrow profile of the principal peak (FWHM < 0.34°) indicates a high degree of crystallinity of this film, i.e., the high quality of the conductive layer.

**Figure 4:**
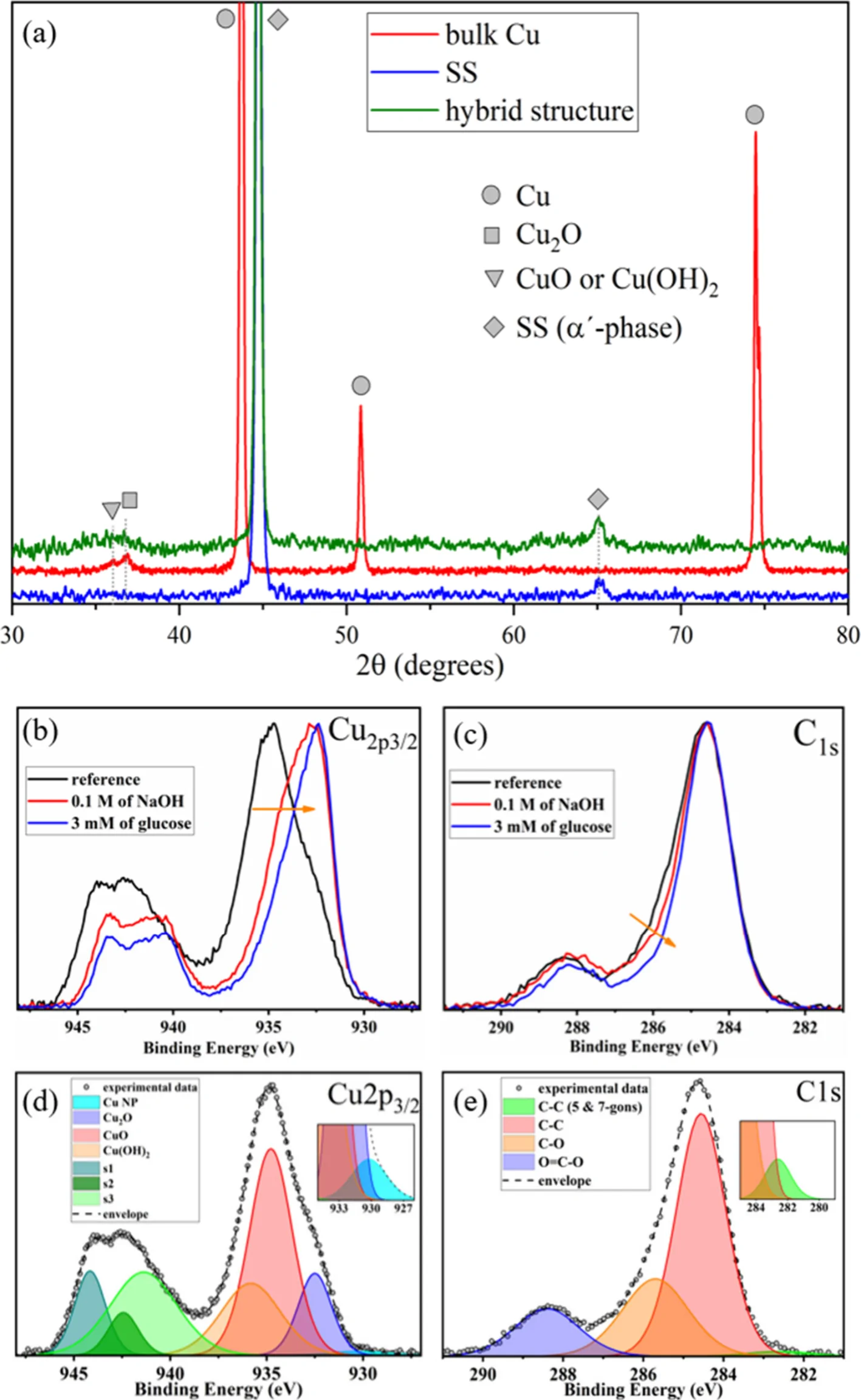
(a) XRD patterns of pure SS and Cu films, and SS/CuONPs hybrid structure. (b, c) Normalized XPS spectra of the Cu 2p_3/2_ and C 1s bands recorded for the as-fabricated sample reference, after electrode testing in 0.1 mol L^–1^ sodium hydroxide and after exposure to 3 mmol L^–1^ glucose, respectively; orange arrows indicate peak shifts (b) or reduction of peak shoulders (c) after testing performance. (d, e) Deconvolution of the XPS spectra of the Cu 2p_3/2_ and C 1s regions, showing the contributions of different copper chemical states and carbon functional groups.

In contrast, the Cu-associated reflexes are not well-defined for the SS/CuONPs structure, with complete absence of signal from metallic copper phase compared to the reflexes at 43.72°, 50. 85° and 74.47° observed in the bulk Cu film, which correspond to (111), (200) and (220) crystalline planes of fcc metallic copper.^45^ However, the weakly resolved peaks near 36.03° and 36.84° attributed to the (111) CuO or the (111) Cu­(OH)_2_ and (111) Cu_2_O planes, respectively,^43,45,46^ which are clearly distinguished from the observed bulk copper sample. In particular, the catalytic activity of copper-based nanomaterials strongly depends on the exposed crystallographic facets since different surface orientations present distinct atomic arrangements. For instance, Cu_2_O nanostructures with dominant (111) facets exhibit enhanced catalytic activity toward glucose oxidation due to their higher density of active sites compared with other crystallographic planes, such as (100).^47,48^ Thus, the diffraction pattern of the SS/CuNPs hybrid structure closely resembles that of the stainless-steel film. The absence of well-defined crystalline features from copper oxide is probably due to the extremely low amount of material related to the CuONPs, causing its signal to be masked by the intense diffraction contribution of the stainless-steel film.

XPS analysis was conducted to investigate the composition and valence state of the elements in the sample. The results (Figure 4 b,c) present a comparison of the Cu 2p_3/2_ and C 1s lines from three samples: as-fabricated reference (S14-ref), treated with 0.1 mol L^–1^ NaOH solution and subjected to 10 cyclic voltammetry cycles at 20 mV s^–1^ (S14–0), and sample treated with NaOH and subsequently used to measure a 3 mmol L^–1^ glucose solution (S14–3), with a baseline correction. All samples exhibit the Cu 2p_3/2_ photoemission peak located between 929.7 and 935.8 eV, along with its corresponding satellite peak at around 940.4 and 944.2 eV (Figure 4 b and d).^49–51^ Deconvolution was performed for all three analyzed samples using Voigt profiles (Figure 4 d and e). Investigation of the main Cu 2p_3/2_ line revealed the presence of three principal components, located near 932 eV, 934 and 935 eV typically associated with Cu_2_O, CuO and Cu­(OH)_2_ chemical bonding, respectively along with a low-intensity component at ∼930 eV attributed to the formation of Cu nanoclusters (CuNPs)^52,53^ In addition, three components were identified in the satellite peak (S1, S2, and S3), positioned near 944 eV, 942 eV and 941 eV, respectively, and attributed exclusively to the Cu^2+^ oxidation state.^49,54^

Following alkaline conditioning (S14-0), the Cu 2p_3/2_ spectrum reveals a marked increase in signal intensity, with the main peak shifted to lower binding energy values, consistent with the presence of Cu^+^/Cu^2+^ species, indicating additional surface oxidation and the formation of Cu_2_O/CuO under alkaline conditions^55,56^ (Figure 4b, Table 1 and Table S1 in Supporting Information). In the S14-3 sample, analyzed after electrochemical glucose detection, a slight shift of the main peak toward binding energies lower than those of the S14-0 sample was observed. This behavior indicates a partial reduction of Cu^2+^ to Cu^+^ species, as during glucose electrocatalytic oxidation, copper oxide species act as an active redox center, and during the conversion of glucose to gluconic acid, Cu^2+^ is reduced to a lower valence state.^27,43^ However, the satellite peaks remain pronounced (940.4–943.4 eV), suggesting the persistence of mixed oxides.

**Table 1:** Results of Quantitative Analysis Obtained from the Analysis of the Cu 2p_3/2_ and C 1s XPS Lines for the Reference, NaOH-Treated, and Glucose-Exposed Samples^a^

Cu 2p_3/2_				
	Relative concentration %			
Sample	Cu NP	Cu_2_O	CuO	Cu(OH)_2_
Reference	1.0	10.7	59.5	28.8
NaOH 0.1 M	0.7	16.3	67.0	16.0
Glucose 3 mM	0.5	20.7	60.9	17.9

C 1s				
	Relative concentration %			
Sample	C–C (5- and 7-gons)	C–C/C–H	C–O or C–OH	O=C–O
Reference	1.6	58.6	24.8	15.0
NaOH 0.1 M	0.6	69.5	10.9	19.0
Glucose 3 mM	2.1	72.8	10.3	14.8

a Relative concentration of each component for the Cu 2p_3/2_ line was calculated considering the contribution of intensities from satellite components. An example of the relative concentration calculation (quantitative analysis) for each copper phase is provided in Section S2 of the Supporting Information.

Quantitative deconvolution of the Cu 2p_3/2_ spectra (see Table S2) revealed a progressive decrease in the Cu^2+^/Cu^+^ ratio from 8.3 for the reference electrode to 5.1 after NaOH conditioning and 3.8 following glucose exposure, as determined from the relative contributions of CuO and Cu­(OH)_2_ (Cu^2+^) and Cu_2_O (Cu^+^). This progressive shift toward a higher relative abundance of Cu^+^ species indicates an evolution of the surface copper oxidation state and is consistent with the participation of Cu^2+^/Cu^+^ redox transformations in the proposed glucose oxidation pathway.

Deconvolution of the C_1s_ line for the three studied samples indicated a principal component at 284.6 eV, attributed to C–C/C–H bonds, along with secondary components at approximately 286.0 eV and at 288.0 eV associated with C–O and O–C=O bonding, respectively (Table 1 and Table S4 in Supporting Information).^57^ In addition, a low-intensity component near 283.0 eV, typically linked to C–C bonds in pentagonal and heptagonal aromatic rings, was identified.

In the S14-ref sample, deconvolution of the C 1s line shows the lowest contribution from C–C/C–H species and the highest proportion of C–O/C–OH bonds, in comparison with the samples treated with NaOH and glucose solution. This is consistent with a highly hydroxylated copper oxide interface, which facilitates the chemisorption or physisorption of oxygenated organic molecules from the atmosphere. After alkaline conditioning (S14-0 sample), a relative increase in the O–C=O species was observed, which can be associated with the formation of surface carbonates resulting from alkaline exposure. This modification coincides with the increased Cu^2+^, as confirmed by the increased CuO component (Table 1) fraction in the Cu 2p_3/2_ spectrum, evidencing that NaOH promotes the oxidation of metallic copper and partial oxidation of the surface carbon layer, resulting in a mixed surface of copper carbonate oxides. In the S14-3 sample, obtained after glucose electrochemical detection tests, the results presented in Table 1 for the C 1s line indicate a decrease in oxygenated species (reduction of the C 1s peak shoulder in Figure 4c) and an increase in the C–C component, suggesting the deposition or adsorption of carbonaceous residues or partially dehydrated products of glucose oxidation, which contribute to electrode surface fouling.

The printed electrode was initially evaluated using cyclic voltammetry (CV) and [Ru­(NH_3_)_6_]­Cl_3_ as a redox probe. Figure 5a shows the cyclic voltammograms obtained by varying the scan rate (20–200 mV s^–1^) for a 5.0 mmol L^–1^ [Ru­(NH_3_)_6_]­Cl_3_ in 0.1 mol L^–1^ Na_2_SO_4._ It is possible to note a well-defined and reversible redox process, typical for this outer-sphere redox probe. The linearity of the relationship between the anodic peak current (Ipa) and cathodic peak current (Ipc) with the square root of the scan rate (ν^1/2^) was confirmed, presenting a coefficient of determination of R^2^ = 0.998 and R^2^ = 0.998, respectively. Therefore, the redox process at the manufactured electrode system is governed by a diffusion process. Using the Randles-Sevcík equation and the diffusion coefficient of [Ru­(NH_3_)_6_]­Cl_3_ reported by Wang et al. (2011),^58^ the electroactive surface area was calculated to be 0.112 cm^2^. Compared with the geometric electrode area of 0.080 cm^2^, this value corresponds to an approximately 40% increase in the active surface area.

**Figure 5:**
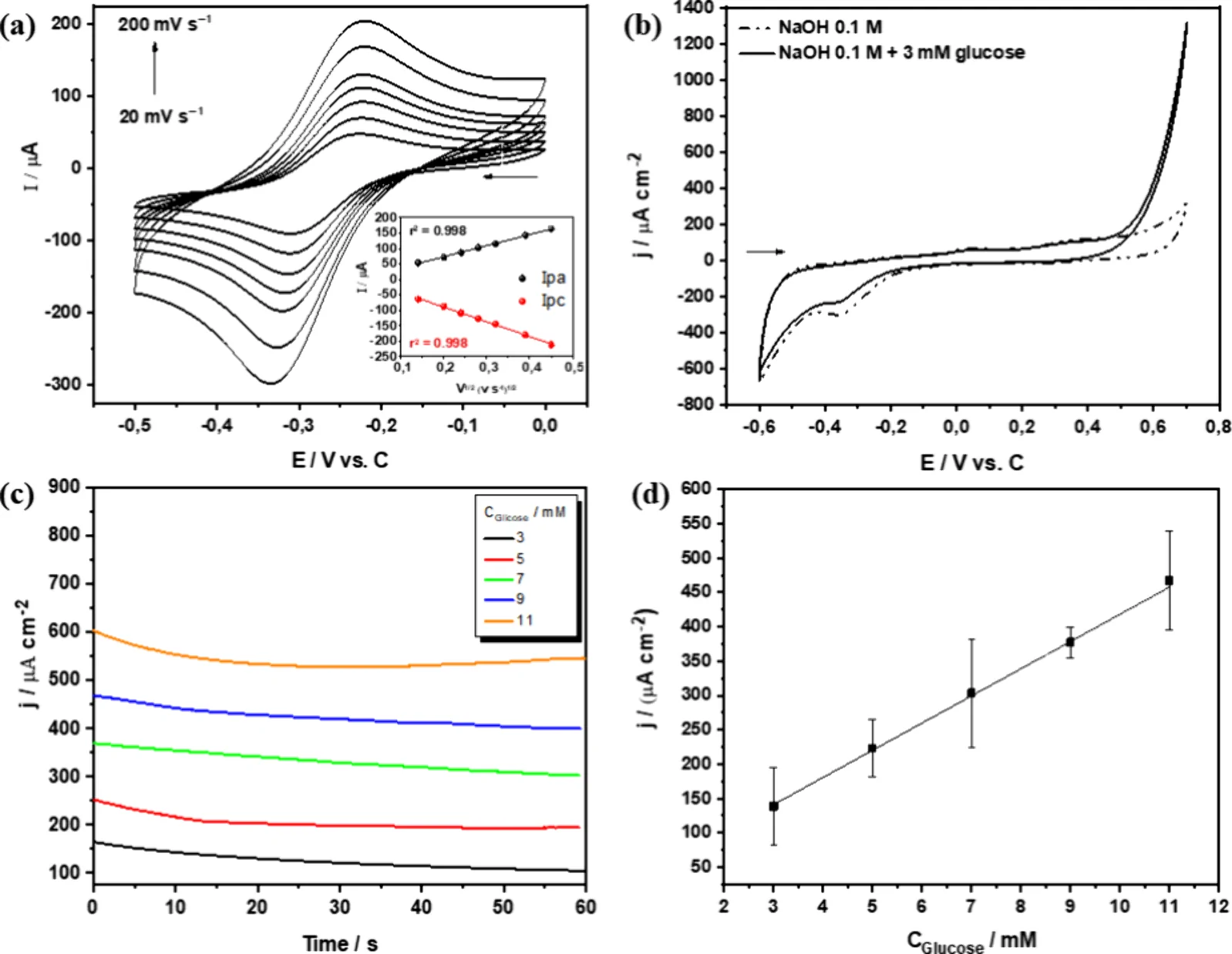
(a) Cyclic voltammograms obtained using different potential scanning rates (20–200 mV s^–1^) in the presence of 5.0 mmol L^–1^ [Ru­(NH_3_)_6_]­Cl_3_ in 0.1 mol L^–1^ Na_2_SO_4_. The inset shows a linear relationship of the anodic and cathodic peak currents as a function of the square root of the potential scan rate. (b) Cyclic voltammograms of the electrodes recorded in 0.1 mol L^–1^ NaOH solution in the absence (dashed line) and in the presence (solid line) of 3 mmol L^–1^ glucose, at a scan rate of 20 mV s^–1^. (c) Chronoamperometric results obtained using the sensor in the presence of different concentrations of glucose: 3.0 mmol L^–1^, 5.0 mmol L^–1^, 7.0 mmol L^–1^, 9.0 mmol L^–1^ and 11.0 mmol L^–1^ in 0.1 mol L^–1^ NaOH solution. Conditions: 0.6 V, 60 s. (d) The respective analytical curve. The error bars represent the standard deviation of 3 measurements.

In order to verify the electrochemical performance of the disposable electrodes toward glucose sensing, the system was also evaluated using cyclic voltammetry (CV). Figure 5b shows the typical CV curves recorded for the electrode in 0.1 mol L^–1^ NaOH solution, in the absence (dashed line) and presence (solid line) of 3 mmol L^–1^ glucose. The voltammetric profile exhibits the characteristic redox transitions of the Cu/Cu_2_O/CuO system in an alkaline medium. The anodic peaks, in the potential range between −0.1 and 0.2 V, can be ascribed to the formation of copper­(I) oxide (Cu_2_O) from metallic copper (Cu^0^) that was not fully oxidized during the thermal treatment as well as to the generation of a mixed layer composed of copper­(II) oxide (CuO) and copper­(II) hydroxide (Cu­(OH)_2_).^47,59^ This pretreatment step is crucial, as it promotes surface homogenization and the formation of a Cu^+^/Cu^2+^ rich layer, which is essential for the electrocatalytic behavior of the sensor. These results corroborate the findings of the XPS study, which showed that alkaline conditioning promotes the formation of a Cu^+^/Cu^2+^ mixed-valence surface. Upon the addition of glucose, a pronounced increase in the anodic current is observed around 0.6 V (Figure 5b), resulting from the catalytic oxidation of glucose mediated by Cu^+^/Cu^2+^. In the reverse scan, the cathodic process observed at −0.3 V corresponds to the reduction of CuO/Cu­(OH)_2_ to CuO_2_.^47,59^

This behavior is consistent with previous reports on copper-based nonenzymatic glucose sensors, in which Cu^+^/Cu^2+^ redox couples play a fundamental role in facilitating glucose oxidation in alkaline media. However, in most of these systems, the formation of electroactive copper species requires additional activation or conditioning steps. In contrast, the methodology proposed here enables direct formation of an electroactive Cu^+^/Cu^2+^ surface, allowing the sensor to be readily employed for glucose detection without the need for prolonged conditioning procedures. This feature represents a practical advantage by simplifying operation. As shown in Table S3, the concentration of copper remains unchanged after alkaline treatment and during glucose tests within the experimental error. This result demonstrates the relative stability of the hybrid structure under glucose sensing conditions. In practical terms, no detectable dissolution of CuO nanoparticles occurs during the sensing performance.

To assess the selectivity of the proposed sensing material for glucose determination in the presence of other relevant biomolecules, a selectivity study was performed using 5 mM glucose and 0.5 mM of potential interfering species, including uric acid, ascorbic acid, dopamine, and fructose, evaluated individually and in combination (Figure S1). Although individual interferents produced measurable current responses under the same experimental conditions, their presence as a mixture did not result in a significant change in the glucose response, even under these conditions designed to simulate a more complex sample matrix. These results indicate that the tested interferents do not significantly affect the electrochemical response toward glucose under the conditions investigated. Furthermore, it is important to note that the physiological concentrations of uric acid, ascorbic acid, and dopamine in biological fluids such as blood are generally lower than those evaluated in this study (e.g., uric acid 120 μM to 450 μM, while dopamine is typically present at nano- to micromolar concentrations).^60,61^

Based on Figure 5b, the amperometric technique was employed for determination purposes due to its simplicity and instrumental similarity with the traditional glucometer’s operation. In this case, the electrode response was assessed in an alkaline medium by applying a constant potential of 0.6 V (vs C) for 60 s using different glucose concentrations (3, 5, 7, 9, and 11 mmol L^–1^). These concentrations are relevant in relation to the physiological levels of glucose found in human blood. In healthy individuals, fasting glucose levels typically range from approximately 3.9 mmol L^–1^ (70.2 mg dL^–1^) to 5.5 mmol L^–1^ (99.0 mg dL^–1^), while postprandial values can increase to around 7 mmol L^–1^ (126.0 mg dL^–1^). In contrast, diabetic patients frequently present with fasting and postprandial glucose concentrations greater than 7.0 and 10.0 mmol L^–1^ (180.0 mg dL^–1^), respectively.

The chronoamperometric results (Figure 5c) exhibit a linear increase in current density (j) with increasing glucose concentration. The current–time curves for all glucose concentrations exhibited quasi-steady-state behavior, characterized by current plateaus, rather than the classical Cottrell response expected for a diffusion-controlled reaction. This behavior suggests that the copper oxide/hydroxide nanoparticles (Cu^+^/Cu^2+^ catalyst species) provide appropriate adsorption strength and confinement of glucose at the active electrode surface, as confirmed by XPS analysis (Figure 4d and Table 1). These surface effects facilitate the electrocatalytic glucose oxidation reaction.^62,63^ The analytical curve is linear within the investigated concentration range (3–11 mmol L^–1^), with an R^2^ = 0.998. The limits of detection (LOD) and quantification (LOQ) were calculated from the linear regression parameters of the analytical calibration curve using the equations LOD = 3­(σ/S) and LOQ = 10­(σ/S), where σ corresponds to the standard deviation of the intercept and S is the slope of the curve.^48^ The obtained values were 0.5 mmol L^–1^ and 1.5 mmol L^–1^. These values confirm adequate sensitivity for glucose detection in clinically relevant concentration ranges. Importantly, unlike many previously reported sensors, the proposed sensor is fabricated without any additional activation or modification steps. Table 2 compares selected analytical parameters of the fabricated electrode with other nonenzymatic glucose sensors.

**Table 2:** Comparison of Electrochemical Sensing Performance for Glucose Detection with Other Nonenzymatic Glucose Sensors

Electrode	Fabrication strategy	Linear range (mM)	Sensitivity (μA mM^–1^ cm^–2^)	LOD	ref
ZnO/CuO/rGO nanohybrid	hydrothermal technique	2 – 10	5660	0.54 μM	^64^
WS_2_/CuO	Liquid-phase exfoliation	0.1–2/2–20	311.23/238.3	0.65 μM	^65^
CuO/rGO nanoflowers	Electrochemical anodization	0.01–3	2294.33	0.9 μM	^66^
CuO/Cu_2_O/SnO_2_ branched supernanowires	Co-precipitation + microwave treatment	-	2112	33.3 μM	^67^
Cu_2_O nanosheets	Diaphragm glow-discharge plasma	0.01–10.2	771	0.15 μM	^68^
Au/ZrO_2_@MWCNTs	Hydrothermal synthesis	0.01–13	162.52	0.95 μM	^69^
NiCu-LDH/CuO/NCC	Hydrothermal + electrodeposition	0.001–1.5	11.545	0.26 μM	^70^
CuO–250	dimethylamine (DMA)-assisted hydrothermal strategy	0.00197–1.39/1.39–7	738.1/104.3	1.97 μM	^71^
Ecoflex:graphite/Au@GQDs	Solvent-assisted method + hydrothermal synthesis	0.1–1.5	4292	9.12 μM	^72^
**SS–CuO**	**DC sputtering + thermal oxidation**	**3–11**	**37.93**	**0.5 mM**	**This work**

Although some recently reported sensors exhibit higher sensitivity and lower limits of detection, such performance is often achieved through the use of more complex architectures, multicomponent nanocomposites, or the incorporation of noble metals. In contrast, the proposed work platform combines a direct and potentially scalable production strategy with a noble metal-free electrode architecture. Furthermore, the proposed sensor exhibits a linear response in the 3 to 11 mM range, covering the physiologically relevant interval of millimolar glucose concentrations. Therefore, the main advantage of the proposed platform lies in the combination of a simple, scalable, and noble metal-free fabrication approach with robust response in the clinically relevant glucose interval, without response saturation, highlighting the practical advantages of this approach compared with methods optimized primarily for high detectability.

## Conclusions

In summary, a simple and scalable fabrication route for a sensory system was demonstrated. The system comprises a stainless steel thin film decorated with copper oxide nanoparticles as the working electrode, and carbon-based films serving as pseudoreference and counter electrodes, all deposited on a flexible PI substrate. Structural characterization using XRD, HRSEM, HR TEM, and SAED confirmed the high quality of the conductive layer as well as the glucose-sensitive CuO nanoparticles. The relatively high proportion of Cu_2_O/CuO fraction in nanoparticle form, verified by XPS and SAED measurements, provides enhanced sensitivity for glucose detection.

Moreover, electrochemical assays performed on this system demonstrated adequate sensitivity for glucose detection within clinically relevant concentration ranges. The combination of scalable, reproducible, and cost-effective fabrication techniques—DC sputtering, screen printing, and low-temperature thermal oxidation—with low-cost stainless steel, copper nanoparticles, and carbon paste enabled the development of an integrated sensing platform with strong practical appeal. Such characteristics make the proposed prototype a promising electrochemical device with potential applications extending beyond the medical field.

These characteristics highlight this fabrication strategy as a promising platform for nonenzymatic glucose detection. Beyond glucose sensing, the versatile production approach and tailored surface properties of these hybrid nanostructures pave the way for broader applications in electrochemical sensing and catalysis, where high activity, stability, and reproducibility are essential.

## Supplementary Material



## Notes

A detailed description of the HR TEM/FFT/SAED image analysis, the treatment of C 1s and Cu 2p_3/2_ XPS peaks, and the results of the selectivity tests (PDF)
