# Supplementary material for: Scalable Fabrication of Stainless Steel–CuO Hybrid Nanostructured Electrodes for Nonenzymatic Glucose Sensing
Source: ACS Omega. 2026 Oct 2;11(40):62072–83. doi: 10.1021/acsomega.6c08394 (PMC13670281; doi:10.1021/acsomega.6c08394)
Supplement: Supplementary file 1 [file ao6c08394_si_001.pdf]

Supporting Information

Scalable Fabrication of Stainless Steel–CuO Hybrid Nanostructured Electrodes for Non-Enzymatic Glucose Sensing

Kelly T. C. Thomaz[1,2], Jordelson A. A. Junior[2,3], Raphael M.V. Rocha[2], Fernando I.L. Leite[2], William Reis de Araujo[1], Andrei Alaferdov[4]*

[1]Laboratório de Sensores Químicos Portáteis, Instituto de Química, Universidade Estadual de Campinas, SP, 13083-862, Brazil.

[2]Department of Hardware and Microelectronics, Instituto de Pesquisas Eldorado, Campinas, SP, 13083-898, Brazil.

[3]Faculty of Electrical and Computer Engineering, Universidade Estadual de Campinas, Campinas, SP, 13083-852, Brazil.

[4]Center for Semiconductor Components and Nanotechnologies, Universidade Estadual de Campinas, Campinas, 13083-870, Brazil.

* Corresponding author

Andrei Alaferdov: E-mail: laferd@unicamp.br

Table of contents

S1. Detailed description of HRTEM/FFT/SAED images analysis…………………..…………...2

S2. Details of C1s and $Cu2p_{3/2}$ XPS peak treatment……………………………………….……..4

S3. Selectivity test towards electrochemical glucose detection……………………………………..7

S1. Detailed description of HRTEM/FFT/SAED images analysis

The data were organized and processed through a Python pipeline with file-level traceability. The structure separates raw data (TEM/HRTEM and SAED images), selected data (the subset used for statistics), processed data (tables and images with detections), and consolidated graphical outputs. An algorithm scans the images and classifies/groups them by sample based on the naming pattern associated with the fabrication parameters, automatically separating the HR (HRTEM) and DIF (SAED) sets.

Nanoparticle Size Analysis (HRTEM)

The lateral size of the nanoparticles was determined from HRTEM images by manually outlining the contour of each visible nanoparticle using a custom Python script. Spatial calibration for each image was obtained from the microscope's scale bar. For each manually outlined contour, the minimum-area circumscribed rectangle was calculated, and the nanoparticle lateral size was defined as the largest dimension of this rectangle (maximum length, analogous to the Feret diameter). The measured values were converted from pixels to nanometers using the corresponding pixel/nm calibration factor.

After confirming the selection, the script: (i) records the particle with an ID and the reference to the source image, (ii) saves an annotated version of the image for traceability, (iii) generates the local FFT of the delimited region and saves each individual FFT with metadata in the filename, and (iv) increments a spreadsheet with the geometric metrics used as the particle size.

For each condition, measurements obtained from multiple HRTEM images were consolidated for N nanoparticles and summarized using descriptive statistics, including the mean, median, standard deviation, standard error, and 95% confidence interval. Nanoparticle size distributions were represented by histograms with bin widths determined using the Freedman–

Diaconis rule, with density curves (e.g., kernel density estimation and normal distribution) overlaid to assess the distribution shape.

Phase identification (SAED and FFT)

Phase identification is performed both on SAED patterns and on the FFTs generated per particle. A script processes each pattern and detects two complementary signatures: Bragg spots (discrete reflections) and Debye–Scherrer rings (approximately concentric, nearly continuous reflections). The algorithm calibrates the pattern scale from each image, calculates the radial distance of each detection from the non-diffracted beam, and converts these distances into experimental d-spacing values, recording them in tables by sample.bThe detections and the cropping/centering steps are saved as images for traceability, and the processing generates log files, summaries, and detailed tables.

Next, the radial detections are grouped into “spheres” (sets with similar distance to the center), and an average d-spacing is calculated for each sphere. Each average value is compared with a theoretical database of interplanar spacings and allowed reflection rules, returning the most compatible phase and (hkl) family/plane within a tolerated error; the result is a table indexed by sample. The microstructural classification is inferred from the diffractometric signature: spheres formed by rings (or rings + spots) are reported as polycrystalline, while spheres formed only by spots are reported as monocrystalline.

Finally, statistical routines aggregate the indexed results and produce three complementary views for the sample: (i) counts of monocrystalline vs. polycrystalline occurrences by phase, (ii) counts of phase combinations observed per particle (among the possible combinations of Cu, $Cu(OH)_2$, Cu_2O, and CuO), and (iii) total phase counts independent of combination, reported as an aggregated fraction according to the metric adopted in the pipeline.

S2. Details of C1s and $Cu2p_{3/2}$ XPS peak treatment

Considering the fact that the satellite peaks located between 944 and 940 eV are exclusively associated with the Cu^{2+} oxidation state [1–5], the relative concentration of each phase can be calculated using equations (1)-(4):

$$c(CuNP) = \frac{A_{CuNP}}{A}; \quad (1)$$

$$c(Cu_2O) = \frac{A_{Cu2O}}{A}; \quad (2)$$

$$c(CuO) = \frac{A_{CuO} + \left(\frac{A_{CuO}}{A_{Cu(OH)2} + A_{CuO}}\right) \times A_S}{A}; \quad (3)$$

$$c(Cu(OH)_2) = \frac{A_{Cu(OH)2} + \left(\frac{A_{Cu(OH)2}}{A_{Cu(OH)2} + A_{CuO}}\right) \times A_S}{A}; \quad (4)$$

where A_{CuNP}, A_{Cu2O}, A_{CuO} and $A_{Cu(OH)2}$ represent the areas associated with the CuNP, Cu_2O, CuO and $Cu(OH)_2$ phases respectively, determined from the main $Cu2p_{3/2}$ peak (Figure 4d, Main text), A_S is the total area associated with shake-up satellite peak components, and A is the total area encompassing all components of $Cu2p_{3/2}$ peak.

Table S1. Detailed results of the deconvolution procedure performed on the $Cu_{2p3/2}$ XPS line for the reference, NaOH-treated, and glucose-exposed samples, reporting binding energies and peak component areas in both absolute and relative values.

Bonding type	Cu NP			Cu_2O			CuO			$Cu(OH)_2$			S1			S2			S3		
Sample	Position (eV)	Area (cps eV)	Area (%)	Position (eV)	Area (cps eV)	Area (%)	Position (eV)	Area (cps eV)	Area (%)	Position (eV)	Area (cps eV)	Area (%)	Position (eV)	Area (cps eV)	Area (%)	Position (eV)	Area (cps eV)	Area (%)	Position (eV)	Area (cps eV)	Area (%)
Reference	930.2	2496	1.0	932.5	26538	10.7	934.8	85386	34.5	935.8	41332	16.7	944.2	25102	10.2	942.4	12546	5.1	941.4	53679	21.8
NaOH 0.1 M	929.9	2165	0.7	932.3	53659	16.3	933.7	142648	43.3	935.5	34054	10.4	943.4	37861	11.6	941.9	12105	3.7	940.6	46477	14.2
Glucose 3 mM	929.7	1632	0.5	932.2	74203	20.7	933.4	144858	40.4	935.1	42628	11.9	943.4	35156	9.9	941.8	17933	5.0	940.4	41651	11.7

Table S2. Quantitative analysis of Cu^{+} and Cu^{2+} species obtained from the deconvoluted Cu $2p_{3/2}$ XPS spectra and corresponding Cu^{2+}/Cu^{+} ratios (considering S1, S2 and S3 satelities peaks).

	Cu^{+} (%)	Cu^{2+} (%)	Cu^{2+} / Cu^{+}
Reference	10.7	88.3	8.3
NaOH 0.1 M	16.3	83.0	5.1
Glucose 3 mM	20.7	78.8	3.8

Table S3. Results of quantitative analysis obtained from three samples using XPS technique.

Sample	Element concentration (at %)						
	C	O	Cr	Fe	Mn	Ni	Cu
Reference	27.4±2.1	45.5±1.7	8.5±1.9	3.9±0.9	3.6±0.9	1.6±0.6	9.4±2.5
NaOH 0.1 M	29.1±3.3	37.9±2.6	12.3±1.9	3.5±0.5	5.0±1.0	0.6±0.2	11.8±2.0
Glucose 3 mM	26.6±1.7	38.8±2.9	12.6±1.4	4.6±0.3	5.2±0.9	0.3±0.2	12.8±2.7

Table S4. Detailed results of the deconvolution procedure performed on the C_{1s} XPS line for the reference, NaOH-treated, and glucose-exposed samples, reporting binding energies and peak component areas in both absolute and relative values.

**Bonding type**	**C-C (pentagons & heptagons)**			**C-C or C-H**			**C-O or C-OH**			**O=C-O**		
**Sample**	**Position (eV)**	**Area (cps eV)**	**Area (%)**	**Position (eV)**	**Area (cps eV)**	**Area (%)**	**Position (eV)**	**Area (cps eV)**	**Area (%)**	**Position (eV)**	**Area (cps eV)**	**Area (%)**
Reference	282.6	624	1.6	284.6	23167	58.6	285.7	9820	24.8	288.4	5911	15.0
NaOH 0.1 M	282.6	304	0.6	284.6	33287	69.5	286.1	5239	10.9	288.1	9084	19.0
Glucose 3 mM	283.2	957	2.1	284.6	33884	72.8	286.0	4710	10.3	288.1	6770	14.8

S3. Selectivity test

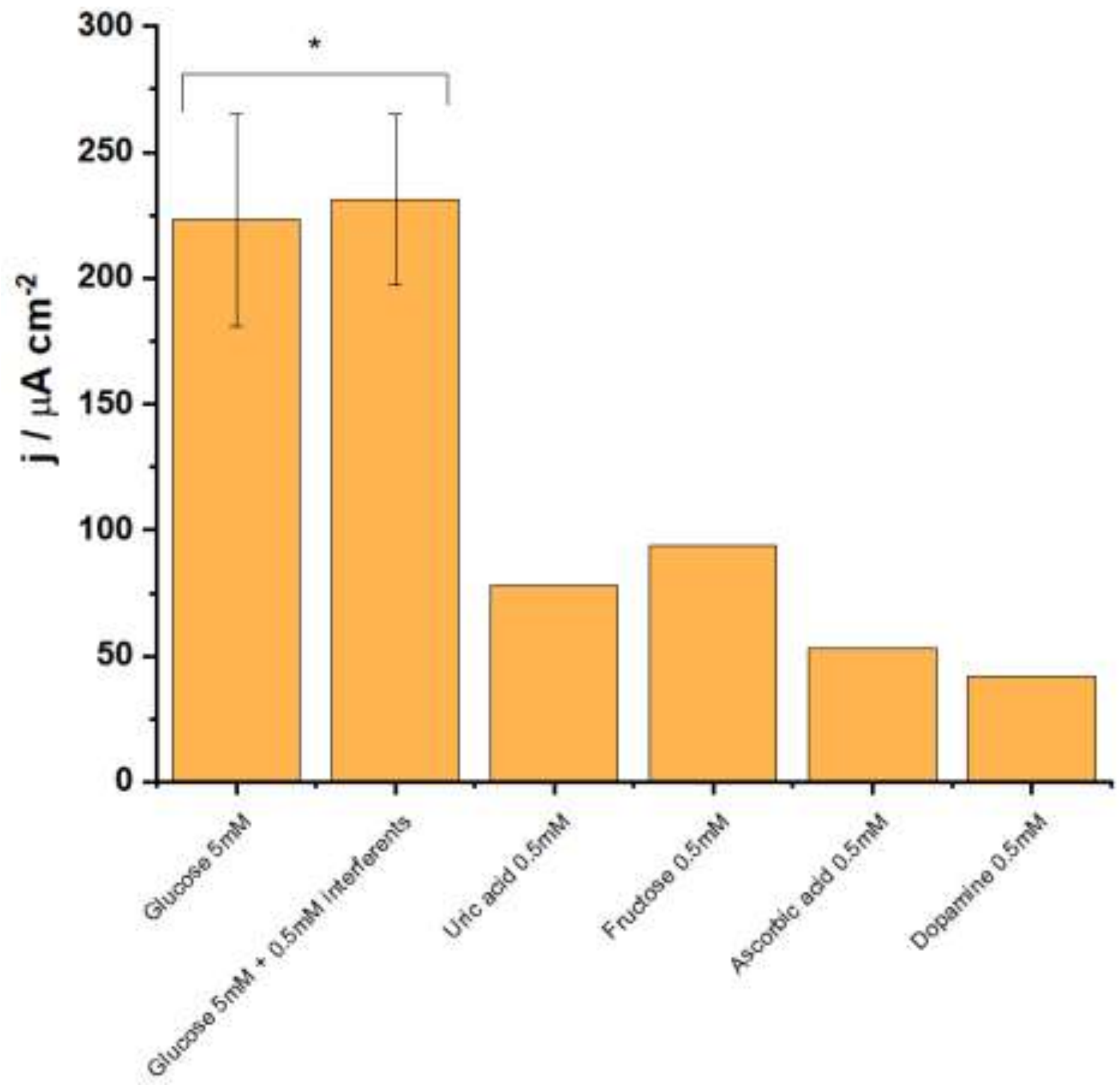

Figure S1. Selectivity test towards electrochemical glucose detection. Current density (j) recorded in the presence of glucose (5 mM), a mixture of 5 mM glucose + 0.5 mM of each potential interfering species evaluated, and individual solutions of uric acid (0.5 mM), fructose (0.5 mM), ascorbic acid (0.5 mM), and dopamine (0.5 mM). Error bars represent standard deviation ($n = 3$). $^*p > 0.05$.

References

(1) Watts, J. F.; Wolstenholme, J. *An Introduction to Surface Analysis by XPS and AES*; Wiley, 2003; Vol. 11. https://doi.org/10.1002/0470867930.

(2) Torres-Ochoa, J. A.; Cabrera-German, D.; Cortazar-Martinez, O.; Bravo-Sanchez, M.; Gomez-Sosa, G.; Herrera-Gomez, A. Peak-Fitting of Cu 2p Photoemission Spectra in Cu0, Cu1+, and Cu2+ Oxides: A Method for Discriminating Cu0 from Cu1+. *Appl. Surf. Sci.* **2023**, *622* (December 2022), 156960. https://doi.org/10.1016/j.apsusc.2023.156960.

(3) Castle, J. E. Use of "Shake-up" Satellites in Photoelectron Spectra for Analysis of Oxide Layers on Metals. *Nat. Phys. Sci.* **1971**, *234*, 93–95. https://doi.org/10.1038/physci234093a0.

(4) Biesinger, M. C. Advanced Analysis of Copper X-Ray Photoelectron Spectra. **2017**, No. February. https://doi.org/10.1002/sia.6239.

(5) Pauly, N.; Tougaard, S.; Yubero, F. Determination of the Cu 2p Primary Excitation Spectra for Cu, Cu2O and CuO. *Surf. Sci.* **2014**, *620*, 17–22. https://doi.org/10.1016/j.susc.2013.10.009.
